# An Approach to Antibody‐Mediated Rejection in Pediatric Liver Transplantation

**DOI:** 10.1111/petr.70455

**Published:** 2026-09-08

**Authors:** Catalina Jaramillo, Parmjeet Randhawa, Amrita Narang, Alison J. Gareau, Jody Weckwerth, Alisha Mavis, George Mazariegos, Katryn Furuya, Nanda Kerkar

**Affiliations:** ^1^ Division of Pediatric Gastroenterology, Hepatology, and Nutrition, Primary Children's Hospital University of Utah School of Medicine Salt Lake City Utah USA; ^2^ Division of Transplantation Pathology, The Thomas E Starzl Transplantation Institute University of Pittsburgh School of Medicine Pittsburgh Pennsylvania USA; ^3^ Section of Transplant Hepatology, Division of Pediatric Gastroenterology, Hepatology, and Nutrition Stanford University School of Medicine Palo Alto California USA; ^4^ Department of Surgery University of Wisconsin‐Madison School of Medicine and Public Health Madison Wisconsin USA; ^5^ Division of Pediatric Gastroenterology and Hepatology Mayo Clinic Transplant Center, Mayo Clinic Rochester Minnesota USA; ^6^ Atrium Health Charlotte North Carolina USA; ^7^ Department of Surgery and Critical Care Medicine, Thomas E. Starzl Transplantation Institute University of Pittsburgh School of Medicine Pittsburgh Pennsylvania USA; ^8^ Division of Pediatric Gastroenterology, Hepatology, and Nutrition University of Wisconsin‐Madison School of Medicine and Public Health Madison Wisconsin USA; ^9^ Division of Pediatric Gastroenterology, Hepatology and Nutrition, Massachusetts General Brigham for Children Harvard University School of Medicine Boston Massachusetts USA

**Keywords:** C4d, donor‐specific antibody, immunosuppression, liver allograft injury, pediatric, plasmapheresis, rejection

## Abstract

Antibody‐mediated rejection (AMR) is an important but infrequent cause of pediatric liver allograft injury. There is a lack of standardized guidance, and treatment approaches vary across institutions. A writing group from the Society of Pediatric Liver Transplantation conducted a comprehensive review of the published literature and collected institutional guidance documents from pediatric transplant centers across the United States to synthesize current diagnostic and management approaches to AMR in pediatric liver transplant recipients. AMR is diagnosed by the presence of characteristic histologic injury, C4d deposition, circulating donor‐specific antibody (DSA), and exclusion of alternative causes of graft dysfunction, including T cell–mediated rejection. Management strategies focus on reducing circulating DSA through therapeutic plasma exchange and suppressing ongoing immune activation with corticosteroids and intravenous immunoglobulin. In severe or refractory cases, targeted therapies such as rituximab, bortezomib, and eculizumab may be considered. A stepwise diagnostic and management algorithm is presented. This resource outlines a practical approach to the diagnosis and management of AMR in pediatric liver transplantation, serving as a reference for busy clinicians who may encounter this serious but underrecognized cause of graft dysfunction and loss.

**Abbreviations**
ADCC: antibody‐dependent cellular cytotoxicity
AI: artificial intelligence
AMR: antibody‐mediated rejection
cfDNA: cell‐free DNA
CR: chronic rejection
Dn: de novo
DSA: donor‐specific antibody
FFPE: formalin‐fixed paraffin‐embedded
HLA: human leukocyte antigen
IgG: immunoglobulin G
IV: intravenous
IVIG: intravenous immunoglobulin
LTx: liver transplantation
MAC: membrane attack complex
MFI: mean fluorescent intensity
miRNA: micro RNA
N:C ratio: nuclear‐to‐cytoplasmic ratio
NK: natural killer
RBC: red blood cells
TCMR: T cell–mediated rejection
US: ultrasound

## 1 Introduction

Antibody‐mediated rejection (AMR) was first described in kidney transplantation in 1997, which led to the development of standardized nomenclature and diagnostic criteria. While these criteria were later adapted for heart, lung, and pancreatic transplantation, the liver was historically viewed as resistant to humoral injury [1]. This “immune‐privileged” status is attributed to several distinct features, including: a vast and stable sinusoidal endothelial surface, the ability of hepatocytes to secrete soluble human leukocyte antigen (HLA) to neutralize antibodies and immune complexes, the presence of Kupffer cells for phagocytosis, and its remarkable regenerative capacity [1, 2, 3].

AMR was first recognized in ABO‐incompatible liver allografts and later found in grafts demonstrating lymphocytotoxic donor‐specific antibodies (DSA) directed at HLA [2]. The increasing evidence of antibody‐mediated liver allograft injury led to the adoption of diagnostic criteria in 2016 [4]. AMR is thought to occur infrequently in pediatric liver transplant (LTx) allografts compared to T cell–mediated rejection (TCMR), which occurs much more frequently, affecting 35% of pediatric LTx recipients in the first year after transplantation [5, 6]. Despite being an important cause of pediatric liver allograft injury, AMR has been poorly described in the literature, leading to a lack of standardized guidance and varying treatment approaches across institutions. We herein review acute and chronic AMR in pediatric LTx recipients, including clinical manifestations, diagnosis, and management.

## 2 Methods

A writing group from the Society of Pediatric Liver Transplantation Education Committee conducted a comprehensive review of the published literature and collected institutional guidance documents from pediatric transplant centers across the United States to synthesize current diagnostic and management approaches to AMR in pediatric LTx recipients. The writing group included pediatric transplant hepatologists, advanced practice providers, transplant surgeons, and pathologists.

## 3 Clinical Presentation

AMR presentation can vary, depending on whether it is acute or chronic. Acute AMR usually presents soon after transplant, sometimes within weeks, and has a rapid onset of liver dysfunction. Patients present with several laboratory abnormalities, including persistently elevated liver enzymes (serum aminotransferases, alkaline phosphatase, gamma‐glutamyl transpeptidase), hyperbilirubinemia, thrombocytopenia not responsive to treatment, and low complement levels (although these are not frequently checked immediately post‐LTx) [7]. The diagnosis of acute AMR is based on the presence of four criteria: (1) histologic features, (2) deposition of complement product C4d, (3) presence of DSA, and (4) exclusion of other causes of the pathologic lesions associated with graft dysfunction, such as TCMR. Thus, acute AMR should always be considered in TCMR not responding to standard therapy. The histopathology of acute AMR is discussed in more detail below, but briefly, it shows microvascular damage, including swollen endothelial cells and dilated capillaries, inflammation, and C4d deposits [8].

Even though acute AMR occurs soon after transplant, there is another category that happens even sooner, hyperacute AMR. Hyperacute AMR is rare and begins immediately after the liver graft has been implanted and can evolve over the subsequent few weeks. It can cause hemorrhagic liver necrosis or lead to hepatic artery and portal vein thrombosis, causing primary graft nonfunction. The histology of hyperacute AMR is generally evident on the explanted liver and shows patches of necrosis, vessel thrombosis, ruptured capsule, and overall enlargement of the graft [7, 8].

Chronic AMR is a slower, indolent process that can develop over months to years. The presentation can be subtle with gradual liver dysfunction or can be silent with normal laboratory values (serum aminotransferases, alkaline phosphatase, gamma‐glutamyl transpeptidase, bilirubin) while developing progressive fibrosis and vasculopathy leading to late‐onset liver failure, making it very difficult to diagnose [7, 9]. The histology of chronic AMR has more fibrosis and vascular injury than acute AMR, but overall, less inflammation, and it can have ductopenia. However, chronic AMR can be difficult to treat and can lead to irreversible graft loss despite increases in immunosuppression [8]. There is no agreed‐upon definition, pathology, or treatment for chronic AMR.

## 4 HLA Antibody Function and Role of DSA

AMR is driven by the production of antibodies against donor HLA antigens on the cell surface. The detrimental role of DSA has been well‐established across solid organ transplantation. Levels of Class I DSA decline sharply post‐LTx [10, 11], which aligns with the liver's ability to absorb it, given its large endothelial surface and constitutive cellular expression of Class I HLA. The liver can also secrete soluble Class I to form immune complexes with DSA that are cleared by Kupffer cells [12], and in this context, DSA does not appear to result in acute graft damage [10]. Initially, it was believed that there was low expression of Class II HLA on the hepatic vasculature, but robust expression has been reported on dendritic cells surrounding the venules, the endothelial cells of the sinusoidal and central veins, and the septal venules, even under noninflammatory conditions [13].

Sensitization to HLA and the development of HLA antibodies occur when a person is exposed to nonself HLA antigens. This occurs through pregnancy, transplantation, or blood transfusion and is initiated when a receptor on the surface of a B cell recognizes its cognate antigen [14]. The development of a humoral immune response begins in the secondary lymphoid organs and culminates in B cell interaction with a CD4^+^ T helper cell, ultimately leading to B cell activation and the production of memory B cells and antibody‐producing plasma cells [14].

DSA can mediate graft injury in multiple ways, including activation of the classical complement cascade and formation of the membrane attack complex, as evidenced by C4d on biopsy [12], recruitment and activation of pro‐inflammatory leukocytes through Immunoglobulin G (IgG) Fc receptor binding, initiating cellular apoptosis, and promotion of endothelial cell dysfunction, which can result in platelet dysfunction and aggregation [14, 15].

### 4.1 Detection of DSA

Testing for DSA is performed by incubating patient serum with polystyrene beads coated with Class I (HLA‐A, B, C) and Class II (HLA‐DR, DQ, DP) antigens. If DSA of the IgG isotype is present, it will bind to the beads and is detected with a fluorescent signal on the Luminex platform. This signal is expressed as the mean fluorescent intensity (MFI) value. HLA laboratories and transplant programs have varying cutoffs for defining positivity, and there can be significant variability between different laboratories with respect to the assay parameters and result interpretation. Many laboratories use cutoffs ranging from 1000 to 2000 MFI as a threshold for assigning positivity, but this often varies based on the organ type. MFI values should be interpreted with caution, given that they are not a direct measurement of DSA strength, and a high MFI does not always predict a positive cellular crossmatch [16]. This can be attributed primarily to the fact that the antigen density on the beads is much greater than would be present on the surface of a cell [17]. This is particularly applicable to DSAs detected against the HLA‐C, DQ, and DP loci, which are HLA antigens expressed at comparatively lower levels in a biological setting. Additionally, these assays can be significantly affected by interference from patient serum, particularly where patients have autoimmune disease, and denaturation of native HLA proteins, resulting in false positivity [17]. This can lead to erroneous DSA results that do not correlate with clinical parameters. If there is suspicion that DSA levels are driving AMR through complement cascade activation, and evidence of C4d deposition on biopsy, a C1q assay can identify complement‐binding DSA. The C1q assay should only detect high‐level DSA and is most useful when interpreted in the context of a biopsy. There is currently no requirement that DSA be quantified in terms of mean fluorescence intensity or titer. However, DSAs with MFI values barely above the reporting threshold should be interpreted with caution and in close collaboration with HLA experts, since these may be an epiphenomenon and even resolve spontaneously [12]. A 2017 study examining LTx recipients showed that 97% of DSA‐positive patients with MFI > 10 000 at 1 year had persistent DSA at 5 years, with 50% of these patients maintaining high levels. Forty percent of patients with MFIs between 1000 and 4999 had a decrease in DSA at 5 years and were considered negative. When MFI is above 1000 risk of graft loss increases [18]. Importantly, MFI remains a semiquantitative, laboratory‐dependent measure and serial comparisons are most meaningful when performed using the same laboratory platform and interpreted with histocompatibility expertise. DSA detection should not be interpreted as synonymous with AMR. Low‐level, isolated, or transient DSA may be clinically silent and may resolve without clear evidence of graft injury, whereas persistent or rising Class II DSA, particularly anti‐DQ/DR DSA with high MFI associated with tissue injury, increases concern for pathogenic humoral alloimmunity [19].

### 4.2 Role of DSA in Pediatric LTx

There is compelling data that in adult liver graft recipients, preexisting Class II DSA is associated with adverse outcomes [12]. Logically, preexisting DSA is far less prevalent in the pediatric population, and pretransplant HLA antibody testing is not performed at all centers, so few studies in adult or pediatric cohorts exist to examine the impact of preexisting DSA alone on outcomes.

While posttransplant monitoring for DSA is not standard practice for most liver allograft patients, there is a growing body of evidence in pediatric patients that Class II de novo (*dn*) DSA does affect long‐term graft survival. In a cohort of 346 recipients, 76 (21.8%) developed *dn*DSA within 2 years posttransplant, with an overwhelming majority (74/76, 97%) of patients producing Class II *dn*DSA only, primarily against DQ antigens. Notably, patients with *dn*DSA had higher incidences of not only AMR, but TCMR and biliary complications [20]. However, *dn*DSA was not associated with graft loss in this patient cohort. These results were consistent with a smaller pediatric study (*n* = 123) showing that of the 54 patients that developed *dn*DSA, 53 (98%) developed a Class II *dn*DSA primarily against DQ antigens, which strongly correlated with fibrosis, biopsy‐proven rejection, and lower graft survival [13]. Wozniak et al. [21] also demonstrated that 94% of recipients who developed *dn*DSA did so against Class II antigens, with DQ *dn*DSA strongly predictive of de novo autoimmune hepatitis and late acute cellular rejection. Taken together, these data indicate that Class II DSA are produced at a much higher frequency post‐LTx and are associated with more severe clinical dysfunction and histological rejection.

Pediatric LTx recipients have distinct immunologic considerations that may influence DSA interpretation. Preexisting HLA sensitization is generally less common in children than in adults because pregnancy and prior transplantation are less frequent. The developing immune system is age‐dependent, and immune monitoring in children must account for maturation of T‐, B‐, and NK‐cells [22] as well as changing infectious and alloimmune risks. In adolescence and during transition to adult care, medication nonadherence and tacrolimus trough variability become particularly important contributors to late rejection and may plausibly promote dnDSA formation [23, 24]. Interestingly, in select pediatric LTx recipients who have undergone complete weaning from immunosuppression, DSA positivity did not indicate unsuccessful operational tolerance [25].

### 4.3 Monitoring Considerations

There is no accepted monitoring strategy for post‐LTx DSA surveillance in pediatrics. In practice, DSA testing is mostly indicated in patients with signs of graft dysfunction. In a clinically stable patient with newly detected DSA, particularly low‐level Class II DSA, immediate therapy for AMR is not indicated in the absence of graft dysfunction or compatible histologic injury. Apractical approach would be to repeat DSA using the same laboratory if able, assess immunosuppression adherence, and follow biochemistries closely. Low‐level DSA can be observed and can be transient. On the other hand, persistent or rising high‐MFI Class II DSA in conjunction with abnormal biochemistries, poor adherence to immunosuppression, or prior rejection should prompt histologic evaluation with a liver biopsy [26, 27].

Protocol or surveillance biopsies may identify subclinical inflammation or fibrosis in stable pediatric LTx recipients. These can be informative for patients in whom immunosuppression withdrawal is being considered or in patients with persistently high DSA. However, this remains a topic of debate and is not uniformly performed across centers [25, 26].

There are no data‐driven, pediatric LTx‐specific recommendations defining how often DSA should be checked after AMR treatment [27]. Therefore, the following intervals should not be interpreted as standardized or evidence‐based recommendations for pediatric liver transplantation. One reasonable pragmatic approach, extrapolated from renal transplantation data and adapted to an individual patient's clinical course, is to obtain DSA at AMR diagnosis and before initiation of antibody‐directed therapy, repeat near completion of therapy, and then follow every 1–3 months until liver biochemistries and DSA improve or stabilize. Once stable, DSA could be spaced to every 3–6 months during the first year post‐AMR treatment, then annually or as needed if clinically indicated [28, 29].

## 5 Histologic Features

### 5.1 Acute AMR

The diagnosis of acute AMR includes the following criteria (1) histologic features, (2) deposition of complement product C4d, (3) presence of DSA, and (4) exclusion of other causes of the pathologic lesions associated with graft dysfunction, such as TCMR (Table 1). A detailed discussion of individual criteria follows.

**TABLE 1:** Banff 2016 criteria for diagnosis of acute AMR in liver allografts.

Diagnostic category	Required criteria
Definite acute/active AMR	All four criteria must be met: Histopathological pattern of injury consistent with acute AMR (e.g., portal microvascular endothelial cell hypertrophy, capillary/venule dilatation, microvasculitis, portal edema, ductular reaction, or cholestasis). Positive serum DSA. Diffuse C4d deposition^a^ (C4d score = 3) on tissue (or portal stromal C4d in ABO‐incompatible allografts). Reasonable exclusion of other insults^b^ causing similar injury patterns.
Suspicious for AMR	Both criteria must be met: Positive serum DSA. Nonzero h‐score with a combined C4d‐score + h‐score of 3 or 4.
Indeterminate for AMR	Criterion 1 and 2, plus either 3 or 4: 1. C4d‐score + h‐score ≥ 2. DSA unavailable, equivocal, or negative. C4d staining unavailable, equivocal, or negative. Coexisting insult potentially contributing to injury.

Abbreviations: AMR, antibody‐mediated rejection; DSA, donor‐specific antibody.

^a^ Optimized C4d staining including positive control is critical for proper evaluation.

^b^ Thrombocytopenia, low serum complement levels, persistence of DSA early after transplantation, and elevated liver injury tests are usually present, but might not be prominent in mild cases.

### 5.2 Histology Scoring (h‐Score)

**FIGURE 1:**
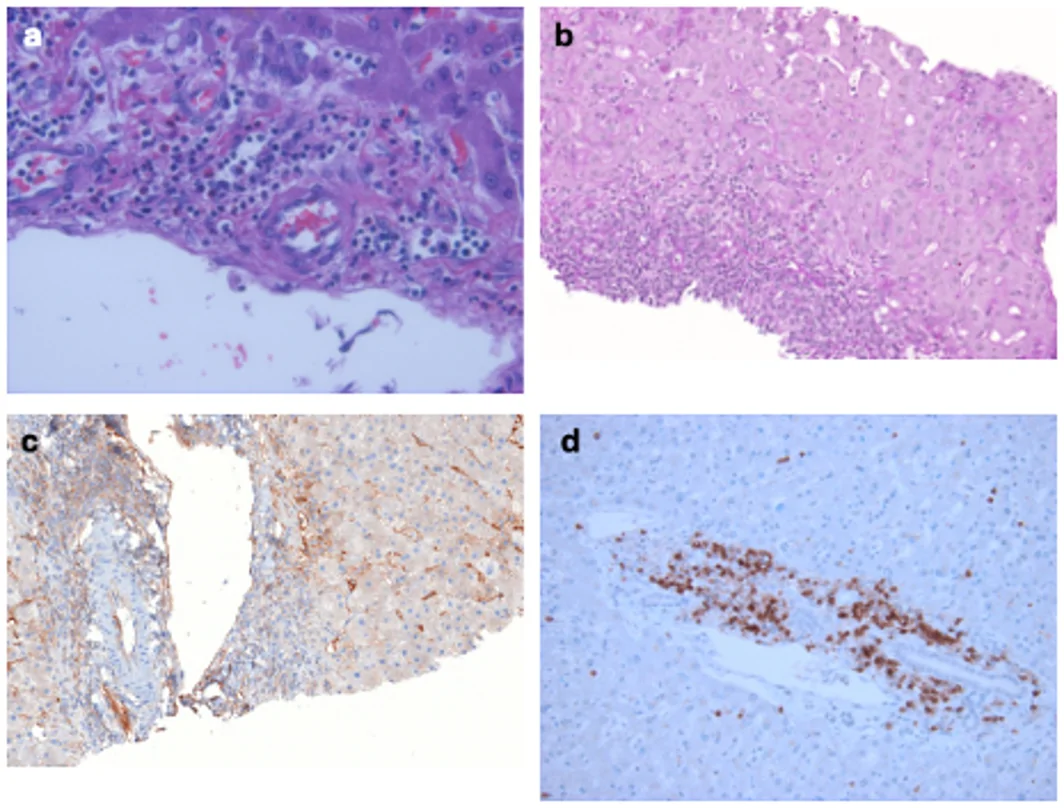
Lesions associated with acute AMR. (a) Hematoxylin eosin‐stained section showing lymphocytes within the portal stromal capillaries (microvascular inflammation). Scattered eosinophils are also seen. (b) Periodic acid Schiff stain highlighting the microvascular basement membranes and showing that microvascular inflammation extends into the periportal sinusoids. (c) Immunohistochemistry for C4d performed on formalin‐fixed paraffin‐embedded tissue. Brown staining decorates capillaries in the portal stroma as well as the basement membranes of periportal sinusoids. Portal vein and hepatic artery endothelium also shows apparent staining, but this finding has low diagnostic specificity. (d) Immunohistochemistry for CD3 showing that the portal inflammatory infiltrate consists primarily of T cells, some of which are causing portal venulitis. This illustrates the important point that most biopsies with AMR have concurrent TCMR, which needs to be treated with steroids or antithymocyte globulin.

Histologic features suggested by the 2016 Banff Working Group are portal and/or microvascular endothelial cell hypertrophy, portal capillary and inlet venule dilatation, monocytic, eosinophilic, or neutrophilic portal microvasculitis, and lymphocytic and/or necrotizing arteritis (Figure 1a,b, Table 2). Fibrin deposition and red blood cell sludging are prominent in ABO‐incompatible allografts with severe injury. Cholestatic features can occur since the arteries and capillaries of the biliary tree are affected [30]. Grading of AMR‐associated histologic lesions is based on the proportion of portal tracts affected and the number of intraluminal inflammatory cells in the most affected capillary (Table 2).

**TABLE 2:** Banff 2016 component lesion scoring for acute AMR.

Score	C4d (immune) score	h (histopathology) score
0	No C4d deposition in portal microvasculature.	No diagnostic features of A\MR present.
1	Minimal: < 10% portal tracts with C4d deposition in > 50% of the circumference of portal microvascular endothelia (portal veins and capillaries).	Portal microvascular endothelial cell enlargement (portal veins, capillaries, and inlet venules) involving a majority of portal tracts with sparse microvasculitis (3–4 marginated and/or intraluminal monocytes, neutrophils, or eosinophils) in the maximally involved capillary; generally mild dilation.
2	Focal: 10%–50% portal tracts with C4d deposition in > 50% of the circumference of portal microvascular endothelia (portal veins and capillaries); usually without extension into periportal sinusoids.	Monocytic, eosinophilic, or neutrophilic microvasculitis/capillaritis (5–10 leukocytes marginated and/or intraluminal) in the maximally involved capillary; prominent portal and/or sinusoidal microvascular endothelial cell enlargement in a majority of tracts/sinusoids; variable but noticeable portal capillary and inlet venule dilatation; variable portal edema.
3	Diffuse: > 50% portal tracts with C4d deposition in > 50% of the circumference of portal microvascular endothelia (portal veins and capillaries); often with extension into inlet venules or periportal sinusoids.	As above, with marked capillary dilatation and marked microvascular inflammation (≥ 10 marginated and/or intraluminal leukocytes in the most severely affected vessels); at least focal microvascular disruption with fibrin deposition and extravasation of RBCs into the portal stroma and/or space of Disse.

Abbreviations: AMR, antibody‐mediated rejection; RBC, red blood cells.

### 5.3 C4d Staining (Immune Staining, i)

C4d deposition is accepted as a surrogate marker for antibody‐mediated injury in all solid organs. It is indicative of activation of the classical or lectin‐binding complement pathways. C4d deposits may occur on the luminal or basal surface of the endothelial cell or on the basement membrane exposed after cell shedding, whereas intra‐sinusoidal serum staining should be regarded as an artifact [31, 32] (Figure 1c). The Banff Schema suggests a semiquantitative scoring system that assigns scores based on the proportion of portal veins and capillaries that stain [4] (Table 2).

C4d staining can be performed in two ways: via immunohistochemistry or immunofluorescence. Most LTx centers use an immunohistochemistry‐based C4d stain performed on formalin‐fixed paraffin‐embedded (FFPE) biopsies for logistical reasons. With this technique, C4d staining localized primarily to the portal microvasculature in most studies [32, 33]. Staining of multiple small capillary channels is more specific than labeling of a larger vein or perihilar bile duct [31, 32]. Not all biopsies with positive staining have Banff AMR lesions, and focal or weak staining may occur in non‐AMR settings. Some show features of ischemia–reperfusion injury, cholestasis, or even hepatitis. Immunofluorescence‐based C4d staining on frozen tissue sections is much more sensitive than immunohistochemistry‐FFPE [32].

Sinusoidal C4d staining has been linked to anti‐angiotensin II receptor type 1 and endothelin type A receptor antibodies [34], but the specificity of this finding is likely low. Angiotensin II type 1 receptor autoantibody levels are higher in liver re‐transplant recipients with graft loss versus those without [35]. However, systematic investigation of antibody levels and the corresponding pathology has not yet been performed across a broad spectrum of patients. Interestingly, another autoantibody directed against glutathione‐S‐transferase has been associated with C4d staining of the portal microvasculature and nerves [36].

### 5.4 Clinical Interpretation of Histologic Acute AMR Scores

Acute AMR can be classified into definitive, suspicious, and indeterminate [4] (Table 1). In practical terms, adjudication of a case as indeterminate or suspicious should only be considered if either the C4d or DSA test is positive. More work is needed to determine the clinical implications of biopsies assigned to the suspicious and indeterminate categories. Experience in kidney transplantation suggests that C4d scores as low as 1 and 2 can reflect significant tissue injury. Indeed, C4d‐negative AMR is a well‐recognized entity in renal allografts. Atypical clinical presentations and biopsy findings need to be kept in mind. A case presenting as sinusoidal obstruction syndrome in the setting of severe central venulitis, no microvascular inflammation, diffuse sinusoidal C4d, and *dn*DSA has been reported [37]. C4d scoring becomes difficult to apply in cases where only focal, weak, and equivocal staining is observed. The rules for scoring also have inherent limitations and cannot adequately capture the broad spectrum of findings seen in clinical practice. Reference to the total clinical picture, including the presence of liver dysfunction, refractoriness to T cell–based treatment, and titers of DSA, can help decide the best course of management in equivocal cases. Experience in kidney transplantation has taught us that AMR should not always be thought of as a binary yes/no disease, but as a spectrum of injury in which some cases demand aggressive therapy, whereas others can be kept under watchful clinical observation [38].

### 5.5 Chronic AMR

**FIGURE 2:**
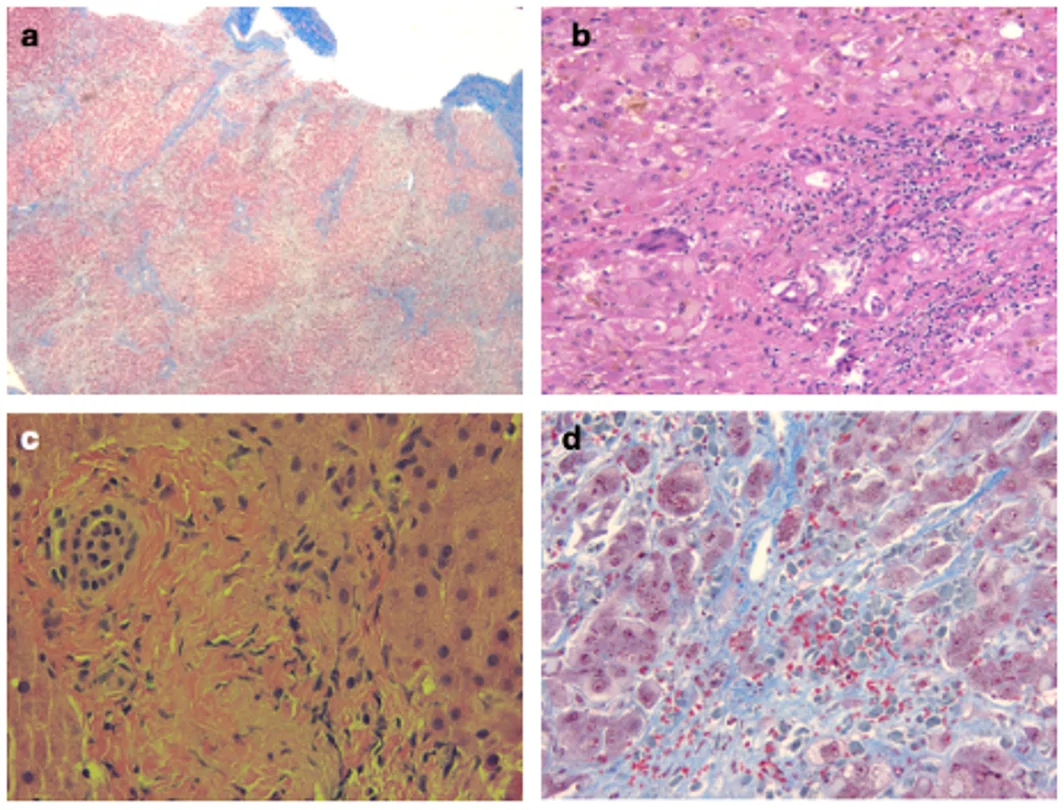
Lesions associated with chronic AMR. (a) A trichrome stain showing portal and periportal fibrosis (collagen fibers are rendered blue). Several foci of portal to portal bridging fibrosis are present. (b) A mild portal mononuclear infiltrate is seen that focally extends beyond the limiting plate (interface activity). The ducts show degenerative changes: the epithelial cells are shrunk (atrophic) with irregular spacing of nuclei that are normally organized into well‐defined circles. The canaliculi are plugged with a yellowish granular material (cholestatic plugging). (c) Some portal triads have dense fibrosis with thick eosinophilic collagen fibers (collagenization of the portal triads). (d) Trichrome stain showing inflammatory cells and extravasated red blood cells around a central vein. This ongoing central venulitis has led to perivenular sclerosis and sinusoidal fibrosis. C4d stain was positive in the capillaries and sinusoids (not shown), and circulating DSA further supported the diagnosis of chronic rejection.

The diagnosis of chronic AMR is a work in progress, and the Banff 2016 Meeting Report only recognizes “probable chronic” and “possible chronic” categories [18, 39] (Table 3). The two histologic criteria include (1) “otherwise unexplained and at least mild mononuclear portal and/or perivenular inflammation with interface and/or perivenular necro‐inflammatory activity” and (2) “the presence of at least moderate portal/periportal, sinusoidal and/or perivenular fibrosis” (Figure 2). A scoring system incorporating these parameters has been shown to have prognostic value [39]. The concurrent presence of DSA and endothelial C4d associates with chronic liver allograft failure [40].

**TABLE 3:** Banff 2016 criteria for chronic active liver allograft AMR.

Diagnostic category	Criteria
Probable chronic active AMR	Requires all four of the following criteria: Histopathological pattern of injury consistent with chronic AMR (both required): Otherwise unexplained and at least mild mononuclear portal and/or perivenular inflammation with interface and/or perivenular necro‐inflammatory activity.^a^ At least moderate portal/periportal, sinusoidal, and/or perivenular fibrosis. Recent circulating HLA DSA in serum samples (e.g., measured within 3 months of biopsy). At least focal C4d‐positivity (> 10% portal tract microvascular endothelia). Reasonable exclusion of other insults that might cause a similar pattern of injury.
Possible chronic active AMR	Clinical and histopathological features as described above, but where C4d staining is minimal or absent.

Abbreviations: ADCC, antibody‐dependent cellular cytotoxicity; AMR, antibody‐mediated rejection; DSA, donor‐specific antibodies; HLA, human leukocyte antigen; TCMR, T cell–mediated rejection.

^a^ It remains challenging to definitively distinguish if mononuclear infiltrates are related to AMR (e.g., ADCC with capillaritis) or TCMR (mostly T effector cells), or a mixed presentation.

While discussing the pathology of chronic AMR, it is important to keep in mind lesions that have been reported as being diagnostic of “chronic rejection” without further qualification with respect to pathogenesis. This list includes duct degeneration, ductopenia, chronic cholangitis, terminal hepatic venule inflammation, perivenular sclerosis, loss of hepatic arterioles, and chronic allograft arteriopathy [41, 42, 43, 44](Table 4). These changes can be the aftermath of T cell or antibody‐mediated injury and may result in biliary strictures or hepatic artery stenosis. Thus, vascular and bile duct problems in LTx are not always the result of technical complications. Immune‐mediated strictures differ from mechanical strictures in that they tend to be proximal to the anastomotic site, are multifocal, and show extension into the intrahepatic branches. Drug‐induced ductopenia, recurrent disease, and ischemic cholangiopathy due to prolonged cold ischemia, reperfusion injury, and vascular flow problems need to be considered in the differential diagnosis. Not infrequently, multiple insults to the biliary tree have occurred, and it is difficult to tease out their relative contributions.

**TABLE 4:** Banff histopathologic staging of chronic liver allograft rejection.

Structure	Early chronic rejection (potentially reversible)	Late chronic rejection (irreversible damage)
	No more than one of the following structures (ducts, venules, or arteries) shows late changes.	At least two or more of the following structures show late changes.
Small bile ducts (< 60 μm)	Degenerative changes in a majority of ducts: Eosinophilic cytoplasmic transformation, increased N:C ratio, nuclear hyperchromasia, uneven nuclear spacing, or ducts only partially lined by epithelium.	Degenerative changes persist in remaining ducts.
	Bile duct loss in < 50% of portal tracts.	Bile duct loss in ≥ 50% of portal tracts.
Terminal hepatic venules and Zone 3	Intimal/luminal inflammation.	Focal vascular obliteration.
	Lytic Zone 3 necrosis and inflammation.	Variable inflammation.
	Mild perivenular fibrosis.	Severe (bridging) fibrosis.
Portal tract hepatic arterioles	Loss involving < 25% of portal tracts.	Loss involving ≥ 25% of portal tracts.
Large perihilar arteries	Intimal inflammation; focal foam cell deposition without luminal compromise.	Luminal narrowing by subintimal foam cells (obliterative arteriopathy); fibrointimal proliferation.
Large perihilar bile ducts	Inflammatory damage and focal foam cell deposition.	Mural fibrosis.
Other findings	So‐called “Transition” hepatitis with spotty hepatocyte necrosis.	Sinusoidal foam cell accumulation; marked cholestasis.

Abbreviations: CR, chronic rejection; N:C ratio, nuclear‐to‐cytoplasmic ratio.

TCMR and AMR‐associated pathology show overlap, and deciding which is the dominant process requires integrated assessment with the clinical and laboratory findings. Notably, perivenular inflammation is mentioned in histologic descriptions of TCMR, and grading of interface activity has been suggested in biopsies with late TCMR [26]. Portal inflammation and portal fibrosis have also been associated with Class II antibodies [45]. Labeling a biopsy as late TCMR does not obviate the need to stain for C4d and test for DSA. Similarly, clinicopathologic correlation is needed to distinguish between immune and nonimmune causes of graft fibrosis, such as portal fibrosis associated with hepatitis C or primary biliary cholangitis, and sinusoidal fibrosis caused by recurrent, de novo, or donor‐derived chronic steatohepatitis and autoimmune hepatitis. Recently, chronic AMR has been associated with nodular regenerative hyperplasia and sinusoidal microvasculitis, defined as the presence of monocyte/macrophage clusters of more than five cells in dilated sinusoids [46]. In essence, this lesion reflects the ability of the liver to respond to a variety of vascular, mechanical, chemical, and autoimmune conditions [47].

Chronic AMR in pediatric LTx should currently be viewed less as a sharply demarcated disease entity and more as a clinicopathologic construct with a broader spectrum. Histopathologic findings should be interpreted in the context of DSA status and trajectory, C4d staining pattern, prior rejection history, immunosuppression exposure and adherence, biliary vascular complications, and recurrent or de novo liver disease. When findings overlap, the interpretation should acknowledge mixed or indeterminate injury rather than forcing a chronic AMR into a binary diagnostic category [26].

### 5.6 Differential Diagnosis

The most frequent cause of graft dysfunction post‐LTx is TCMR. Vascular and biliary causes should be ruled out. There can also be recurrence of disease post‐LTx, including recurrent autoimmune hepatitis and recurrent sclerosing cholangitis. There is additionally a rare form of graft dysfunction in pediatric LTx recipients that resembles AIH, in patients not transplanted for AIH, known as *dn*AIH [48, 49]. In adults, this has been classified as plasma cell rejection [4, 50]. In biopsies where a diagnosis of AMR can be made, it is usually possible to demonstrate lesions conventionally associated with TCMR, such as portal vein endothelialitis, duct damage, and central venulitis [51]. This is because the T cell and antibody‐mediated limbs of the human immune system frequently work in a coordinated fashion. Biopsies with these findings should be designated as mixed TCMR and AMR (Figure 1d). The continuum of alloimmune activation encompassing TCMR and AMR is emphasized by transcriptomics studies showing that subclinical TCMR with DSA positivity is associated with higher expression of inflammation and fibrosis‐associated genes than DSA‐negative biopsies [40]. It is important to recognize that intracapillary inflammation can be seen in many diseases, including viral hepatitis, autoimmune hepatitis, primary biliary cholangitis, primary sclerosing cholangitis, and adverse drug reactions. H‐scoring for microvascular inflammation is not indicated in these biopsies.

## 6 Management

There are no formal guidelines for the management of AMR in pediatric LTx, and high‐quality clinical trials in both pediatric and adult LTx populations are lacking. The therapeutic evidence base for AMR in pediatric LTx remains low. Current treatment strategies are varied and extrapolated from adult liver and kidney transplant literature [4, 8, 52, 53]. Thus, the information in this document should not be interpreted as standardized therapies for AMR in pediatric LTx.

**FIGURE 3:**
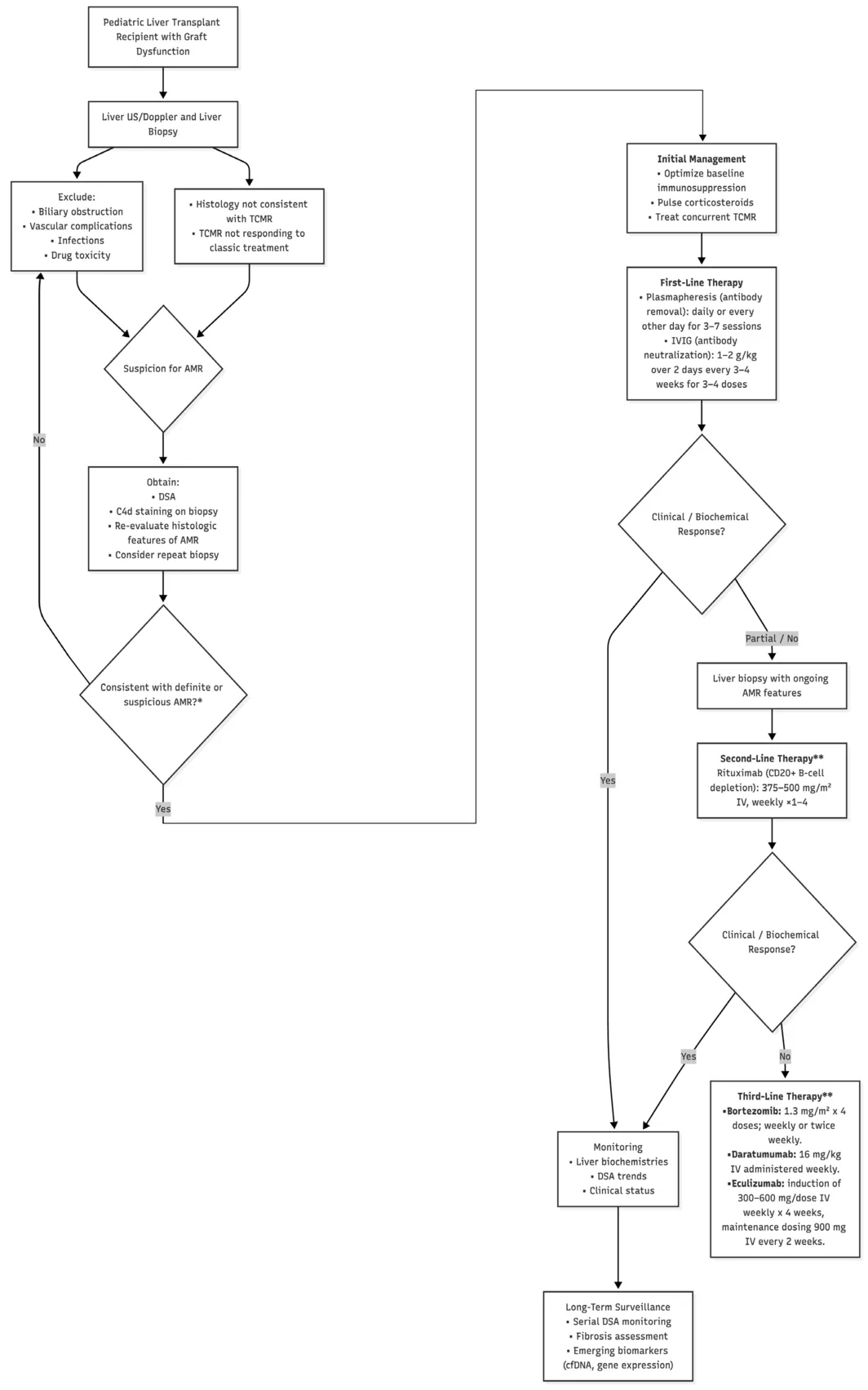
Management algorithm for suspected and confirmed antibody‐mediated rejection (AMR) in pediatric liver transplantation. This algorithm provides a pathway for the diagnosis and therapeutic intervention of antibody‐mediated rejection (AMR) in liver allograft recipients. *See Table 1 for acute AMR and Table 3 for chronic AMR diagnostic criteria. **Second‐ or third‐line therapies can be considered earlier on in select cases with severe presentation where biopsies indicate “definite AMR.” AMR, antibody‐mediated rejection; cfDNA, cell‐free DNA; DSA, donor‐specific antibody; IV, intravenous; IVIG, intravenous immunoglobulin; TCMR, T cell–mediated rejection; US, ultrasound.

Foundational therapies for AMR include plasma exchange, intravenous immunoglobulin (IVIG), and corticosteroids to reduce circulating DSAs and suppress immune activation [52, 53]. In refractory or severe cases, additional immunomodulatory agents such as rituximab and bortezomib may be added [54, 55, 56](Table 5). Other emerging therapies, such as IL‐6 antagonists (tocilizumab, clazakizumab) and complement inhibitors (eculizumab), are under investigation in other transplant populations but lack data in LTx [57]. Ultimately, AMR management in pediatric LTx recipients remains highly individualized, guided by adult literature and tailored to the unique immunologic considerations of children. A suggested approach is illustrated in the algorithm (Figure 3).

**TABLE 5:** Therapy options for management of AMR.

Medication	Mechanism of action	Dosing and frequency
IVIG	Immune modulation: Neutralizes circulating antibodies and blocks the receptors that allow them to attack the graft.	1–2 g/kg given IV over 2 days every 3–4 weeks for 3–4 doses. Most center give initial dose after plasmapheresis completion.
Plasmapheresis	Mechanical removal: Direct “washout” of circulating DSAs from the plasma.	1–1.5 plasma volumes per session for 3–7 sessions, typically performed every other day.
Rituximab^a^	B cell depletion: Anti‐CD20 antibody that removes B cells before they can mature into antibody‐secreting plasma cells.	375 mg/m^2^ IV single dose or repeated weekly for 3–4 weeks. Higher doses of 500 mg/m^2^ have also been reported. Wait 48–72 h after the last plasmapheresis session before giving Rituximab.
Bortezomib^b^	Proteasome inhibitor: Triggers apoptosis (cell death) in plasma cells by causing a buildup of toxic proteins within the cell.	1.3 mg/m^2^ IV doses × 4 doses given weekly or twice weekly. Administer after the plasmapheresis session on treatment days.
Daratumumab^a^	Anti‐CD38 antibody: Directly depletes long‐lived plasma cells and NK cells, striking the actual source of the antibodies (DSAs). Emerging salvage therapy.	16 mg/kg IV weekly for 4 weeks. Administer after the plasmapheresis course is complete. If given during plasmapheresis, redosing is required after the session.
Eculizumab^a^	Complement inhibitor: Binds to C5, preventing its cleavage into C5a/C5b. This stops the formation of the MAC, which otherwise “punches holes” in graft cells. Emerging salvage therapy.	Induction phase: 300–900 mg/dose IV weekly for 4 weeks Maintenance: 300–1200 mg IV every 2 weeks. Dosing based on pediatric weight ranges. Dose must be repeated immediately after a plasmapheresis session. If given as a standalone, do not perform plasmapheresis for at least 24–48 h post‐dose.

Abbreviations: AMR, antibody‐mediated rejection; DSA, donor‐specific antibodies; IV, intravenous; IVIG, intravenous immunoglobulin; MAC, membrane attack complex; NK, natural killer.

^a^ These are large IgG‐based molecules that reside almost entirely in the intravascular space initially and are removed by plasmapheresis.

^b^ This is a small molecule that distributes rapidly into tissues. Giving it after plasmapheresis ensures maximum serum concentration.

In adult LTx recipients, Dumortier et al. conducted a nationwide retrospective study of 47 adults with biopsy‐confirmed AMR and found that the most used regimen, plasmapheresis + IVIG + rituximab, was employed in 56.8% of cases. Despite aggressive treatment, patient survival at 1, 5, and 10 years was 77%, 55.9%, and 55.9%, respectively, while graft survival at the same intervals was 69.5%, 47.0%, and 47.0% [58]. In another retrospective, multicenter study, Sakamoto et al. [54] described 13 patients (5 pediatric, 8 adult), noting limited benefit from rituximab monotherapy; only 2 of 4 children with chronic AMR showed improvement, and adults with acute AMR had poor outcomes, including two deaths despite treatment. A single‐center case series by Wu et al. [55] included 13 patients (11 pediatric, 2 adult LTx recipients) and demonstrated normalization of liver function in 92.3% following treatment with high‐dose corticosteroids, IVIG, rituximab, and bortezomib, though long‐term graft outcomes were unclear. Additional case series have described variable outcomes with multimodal regimens involving corticosteroids, plasmapheresis, IVIG, rituximab, bortezomib, antithymocyte globulin, and mycophenolate mofetil [52, 56].

### 6.1 IVIG

IVIG is frequently used, either alone or as part of combination therapy, for AMR in pediatric LTx. Its exact mechanism of action is not fully defined, but proposed immunomodulatory effects include neutralization of alloantibodies, upregulation of inhibitory FcγRIIB receptors on B cells, and inhibition of complement activation [59, 60]. Dosing regimens vary, with some centers using low‐dose IVIG (100–500 mg/kg intravenously) and others employing high‐dose IVIG (1–2 g/kg administered over 2 days every 3–4 weeks for 3–4 doses). Adjustments are typically guided by clinical response and changes in donor‐specific antibody titers. Low‐dose IVIG is usually combined with other modalities such as plasmapheresis or rituximab. Premedications, including acetaminophen, antihistamines, or corticosteroids, are often administered to minimize infusion‐related reactions. IVIG is generally well tolerated, with most adverse reactions limited to mild infusion‐related symptoms, although rare but serious complications such as renal dysfunction, thrombosis, or hemolysis can occur. While pediatric‐specific data remain limited, IVIG has been associated with reduced rejection episodes and improved graft outcomes, supporting its role as a key adjunct in AMR management.

### 6.2 Plasmapheresis

Plasmapheresis (therapeutic plasma exchange) is a mainstay therapy for AMR, functioning through the physical removal of circulating DSA and other pathogenic plasma proteins that drive alloimmune injury. Protocols typically involve 1–1.5 plasma volume exchanges per session, performed daily or every other day for 3–7 sessions, with treatment duration guided by clinical severity, liver function tests, and changes in DSA titers [52, 61, 62]. It is rarely used alone and is usually combined with IVIG, corticosteroids, and, in more resistant cases, rituximab or bortezomib [62]. Monitoring includes serial DSAs and liver function tests. It requires central venous catheterization, which increases the risk of infection and thrombosis. Younger children may be more vulnerable to hemodynamic instability and metabolic complications due to smaller blood volumes. Although pediatric‐specific data are limited, case series and extrapolation from other solid organ transplants suggest that plasmapheresis, when initiated early and used in combination regimens, improves short‐term graft function and reduces DSA titers, though recurrence of AMR and progression to chronic rejection remain challenges [52, 61, 62].

### 6.3 Rituximab

Rituximab is employed as adjunctive therapy for AMR, particularly in refractory or severe chronic AMR. It is a chimeric monoclonal antibody and acts through depletion of CD20^+^ B cells, thereby reducing production of DSA that drives AMR. Rituximab induces B cell lysis via antibody‐dependent cellular cytotoxicity, complement‐mediated cytotoxicity, and apoptosis [1]. Pediatric dosing typically mirrors protocols from other solid organ transplants. (Table 5) [62, 63]. Premedication with corticosteroids, acetaminophen, and antihistamines is standard to reduce infusion‐related reactions. Monitoring includes serial CD19^+^/CD20^+^ B cell counts, DSA titers, liver function tests, and surveillance for cytopenia and infectious complications. Adverse effects include infusion reactions (fever, chills, hypotension), cytopenia, and increased risk of infections, particularly in the first 3 months post‐administration, while rare but serious risks include progressive multifocal leukoencephalopathy and severe mucocutaneous reactions [59, 62]. Rituximab is not recommended as monotherapy; rather, it is combined with other immunomodulatory agents. Data in pediatric LTx are limited, but case series and a Japanese multicenter study reported biochemical and immunologic improvement in some children with chronic AMR, though outcomes in acute AMR were less favorable, and graft failure still occurred despite therapy [54, 62, 63].

### 6.4 Bortezomib

Bortezomib, a reversible 26S proteasome inhibitor originally approved for multiple myeloma, has emerged as an adjunctive therapy for AMR in solid organ transplantation due to its ability to target plasma cells—the primary source of DSA production. By disrupting protein degradation pathways, bortezomib induces plasma cell apoptosis, inhibits NF‐κB activation, and thereby reduces alloantibody secretion and downstream inflammatory signaling. Data supporting bortezomib for AMR in pediatric LTx are extremely limited, with no dedicated pediatric liver AMR series published to date; its use is extrapolated from adult liver AMR case series and pediatric kidney transplant experience, where plasma cell depletion has been associated with reductions in DSA and short‐term graft improvement. Dosing is also extrapolated from adult LTx or kidney transplant protocols (Table 5) and is often given in combination with plasmapheresis and IVIG [52, 56, 64]. It can be administered both intravenously and subcutaneously with equivalent efficacy, but lower rates of peripheral neuropathy have been reported with SQ dosing [65]. Monitoring includes serial DSA levels, liver function tests, complete blood counts, and neurologic assessment for early signs of neuropathy. While generally well tolerated, side effects include gastrointestinal upset, fatigue, transient thrombocytopenia (with nadirs around Day 11 and recovery within 10 days), and, most significantly, dose‐related peripheral sensory neuropathy. Severe myelosuppression and infectious complications are less common but require vigilance.

### 6.5 Daratumumab

Daratumumab, a human monoclonal anti‐CD38 antibody known for multiple myeloma therapy, is being used off‐label as a plasma cell‐directed therapy for refractory AMR in solid organ transplantation. CD38 is highly expressed on antibody‐producing plasma cells, and daratumumab induces their depletion through complement‐dependent cytotoxicity, antibody‐dependent cellular cytotoxicity, phagocytosis, and apoptosis, thereby targeting the source of ongoing DSA production. Dosing in solid organ transplant AMR is not standardized but generally mirrors oncology (Table 5). Safety considerations include infusion‐related reactions, cytopenias, and increased risk of infection, particularly viral infections, as well as the potential for hepatitis B reactivation, necessitating pretreatment screening and posttreatment surveillance. Data in pediatric LTx are limited to isolated reports, including a case of refractory AMR following ABO‐incompatible deceased donor LTx in which four weekly doses of daratumumab led to biochemical, histologic, and immunologic improvement after failure of plasma exchange, IVIG, and steroids [66]. Broader pediatric experience comes from kidney and heart transplantation [67, 68]. In pediatric heart transplantation, a single‐center series of nine patients treated with daratumumab for AMR reported improvement in all AMR cases, and repeated infusions were also used for chronic AMR [68].

### 6.6 Eculizumab

Eculizumab, a humanized monoclonal antibody targeting complement protein C5, has been explored as a therapeutic option for AMR in pediatric LTx due to its ability to inhibit terminal complement activation, thereby preventing the formation of the membrane attack complex (C5b‐9) and reducing complement‐mediated graft injury. There are no randomized controlled trials or formal guidelines supporting eculizumab for AMR in pediatric LTx, and its use is based on extrapolation from a single case report in pediatric LTx [69] and other solid organ transplant settings [70, 71]. Standard dosing is extrapolated from hematology/renal indications, beginning with an induction phase followed by maintenance dosing. (Table 5). Because eculizumab impairs complement‐mediated bacterial defense, particularly against *Neisseria meningitidis* , patients require meningococcal vaccination at least 2 weeks prior to therapy or appropriate antibiotic prophylaxis if urgent initiation is needed. Monitoring includes assessment of DSA levels, complement activity assays (e.g., CH50), liver function tests, and vigilance for breakthrough infections. Adverse effects are generally mild, including headache, nausea, hypertension, and infusion reactions; however, the most clinically significant risk is life‐threatening meningococcal sepsis, making stringent infection prophylaxis and monitoring critical in the transplant population.

## 7 AMR in Other Solid Organ Transplantation

### 7.1 Heart AMR

Risk factors for death in patients awaiting heart transplants include a limited donor pool and sensitization. The latter refers to the development of immunity against HLA. Formed HLA antibodies can later target antigens on the transplanted organ, leading to graft dysfunction. Hence, the presence of HLA antibodies before heart transplantation is a risk factor for early allograft rejection, graft loss, and death [1]. AMR has a prevalence of 8% and is associated with higher graft loss when compared to ACR in the first year post‐heart transplantation [72].

AMR in heart transplantation is characterized by clinical signs of allograft dysfunction alongside histological and/or immunopathological evidence of rejection on endomyocardial biopsy scored according to the International Society for Heart and Lung Transplantation grading system [73]. Recent data from the Pediatric Heart Transplant Society describe therapeutic practices with up to 50% of patients receiving IVIG, rituximab in 39%, bortezomib in 13%, and eculizumab in 4%. Basiliximab, daratumumab, and alemtuzumab were used rarely. Notably, there was significant treatment variation [72].

### 7.2 Kidney AMR

AMR following kidney transplantation is the leading cause of graft failure with an estimated incidence of 5% [74]. The definition of AMR in kidney transplantation is complex and broadly categorized into definite AMR, probable AMR, and microvascular inflammation that is negative for both DSA and C4d according to the Banff Classification of Renal Allograft Pathology. Diagnosis is based on a combination of three key findings: evidence of microvascular inflammation on biopsy, deposition of complement component C4d, and serologic evidence of circulating DSA [75].

Treatments used for kidney AMR are based on adult desensitizing protocols for the reduction of preexisting anti‐HLA antibodies in highly immunized patients. IVIG, rituximab, and plasmapheresis are the most frequently used [74]. According to the 2019 Expert Consensus from the Transplantation Society Working Group, the recommended standard of care for early (< 30 days posttransplantation) active AMR and late (> 30 days posttransplantation) active AMR with preexisting DSA is plasmapheresis, steroids, and IVIG. Whereas late chronic AMR and AMR with *dn*DSA is treated with optimization of baseline immunosuppression. Adjunctive therapy with rituximab, complement inhibitors, and splenectomy should be considered in early AMR.

## 8 Recent Advances

AMR is a significant cause of graft loss in organ transplantation. The diagnosis and effective management of AMR remain elusive. Monitoring graft function in transplant recipients using noninvasive methods is a field where a host of new strategies are being developed. Donor‐derived cell‐free DNA (cfDNA), consisting of DNA fragments released into the circulation during apoptosis or necrosis, has emerged as a noninvasive biomarker for allograft injury [76]. In a study using over 1000 samples from cardiac transplant recipients, cfDNA, donor‐specific fraction (DF) was used as a direct marker of organ injury [77]. The authors had previously correlated elevated DF with acute cellular rejection and AMR in both pediatric and adult heart transplant recipients but found limitations related to cost and sensitivity. Serial elevation of the donor‐specific fraction of cfDNA has been correlated not only with liver graft injury secondary to rejection but also found to be elevated before rejection develops, thus acting as a biomarker to guide immunosuppression strategies to optimize graft function [78]. Levitsky et al. [79] have reported a genomic profile in LTx recipients with stable graft function versus rejection, and developed a blood‐based biomarker that can be detected before acute rejection develops, which is distinct from the pattern seen in LTx recipients who have not developed acute rejection.

Micro RNA (miRNA) represents another promising frontier in noninvasive diagnostics. miRNA inhibits translation of its complementary target mRNA, thereby controlling gene expression. More than 2000 miRNAs have been identified in serum, plasma, urine, and saliva. A single miRNA can inhibit the transcription of several mRNAs, thus significantly silencing their expression. Matz et al. [80] report their experience in developing a biomarker for AMR using miRNA in kidney transplant recipients but had difficulty establishing specificity for a single pathology with confounding factors like associated fibrosis and infections. They also explored using miRNA as a tool to better understand the molecular mechanisms that lead to the pathogenesis of AMR.

The evolving understanding of the molecular landscape not only informs diagnosis but also highlights the need for a parallel evolution of therapeutic options. Currently, there is an unmet need for therapeutic drugs that can effectively manage AMR. In a recent review, Heeger et al. [81] discussed advances made using innovative approaches, including targeting interactions between alloreactive T cells and B cells, and depleting alloreactive memory B cells, as well as donor‐specific antibody‐producing plasma blasts and plasma cells. Therapeutic options for AMR are expanding, ranging from existing to evolving therapies, including IL‐6/IL‐6R signaling inhibitors (clazakizumab and tocilizumab) and novel agents like the C1 esterase inhibitor imlifidase. Given the heterogeneity of AMR, better phenotyping of patients will likely allow the best agent or combination of therapeutic agents early to allow improved graft function and survival.

As the availability of biomarkers and therapeutic targets grows in AMR, the complexity of clinical decision‐making increases. Studies have shown that artificial intelligence (AI) and machine learning can outperform traditional histology in predicting cardiac transplant rejection [82] and have the potential to improve the accuracy of histopathological diagnosis in a noninvasive manner [83]. AI is a rapidly evolving technology with future potential to impact how we diagnose and manage AMR. As with any new technology, it is essential that findings are validated in multicenter studies with diverse populations and that ethical standards are maintained.

## 9 Summary and Conclusions

AMR is being increasingly recognized in LTx, including pediatric LTx. Although TCMR remains more prevalent, AMR represents an important and underappreciated cause of graft dysfunction, morbidity, and mortality. It is therefore important to consider AMR when one encounters graft dysfunction, particularly when there is an inadequate response to standard therapy for TCMR. The diagnosis of acute AMR is based on the presence of four criteria, including characteristic histologic features, evidence of complement activation such as C4d deposition, presence of DSA, and exclusion of other etiologies of graft dysfunction. Chronic AMR remains less clearly defined, underscoring the need for further refinement of diagnostic criteria and standardized definitions in this setting.

Management strategies for AMR are centered on reducing circulating DSA by therapeutic plasma exchange and suppressing ongoing immune activation with immunosuppression. Therapeutic plasma exchange, IVIG, and corticosteroids form the cornerstone of first‐line therapy, while additional targeted agents, including rituximab, bortezomib, and eculizumab, may be considered in refractory or severe cases. We have developed a pragmatic, stepwise management algorithm derived from the existing literature and clinical practices across multiple pediatric transplant centers in the United States (Figure 3).

As the field of transplantation continues to evolve, emerging advances in immunologic monitoring, artificial intelligence, and machine learning hold promise for earlier detection, risk stratification, and individualized management of AMR. With continued progress in these areas, there is optimism that antibody‐mediated injury can be increasingly prevented, promptly identified, and more effectively treated, ultimately improving long‐term graft and patient outcomes following LTx.

## Funding

The authors have nothing to report.

## Disclosure

Dr. Kerkar is on the Advisory Board for Mirum and Orphalan. She receives royalties from Elsevier.

## Data Availability

Data sharing is not applicable to this article as no datasets were generated or analyzed during this study.
